# Ultrasound Detection of Human Botfly Myiasis of the Scalp: A Case Report

**DOI:** 10.7759/cureus.11905

**Published:** 2020-12-04

**Authors:** Chad H Jones, Marino Leon, Jena Auerbach, Jessica Portillo-Romero

**Affiliations:** 1 Internal Medicine, University of Florida Health, Gainesville, FL, USA; 2 Pathology, University of Florida Health, Gainesville, FL, USA

**Keywords:** myiasis, botfly, ultrasound, dermatobia hominis

## Abstract

*Dermatobia hominis*, also known as the human botfly, is an insect native to Central and South America that is known to parasitize both human and animal hosts through cutaneous infestation by its developing larvae. While human botfly myiasis has been commonly diagnosed through dermatologic findings, the presenting lesions and associated symptoms can be non-specific and often misconstrued as other more common cutaneous diagnoses. Here, we present a case of botfly myiasis of the scalp in which ultrasound was utilized to visualize the larvae and confirm the diagnosis prior to larval removal. In this report, we discuss our patient’s presentation, ultrasound imaging, and clinical course/treatment in order to convey how ultrasound imaging, when available, is a valuable tool in establishing the diagnosis of human botfly myiasis.

## Introduction

The process of myiasis occurs when humans or vertebrate animals are infested by fly larvae, which grow inside the host and feed on its tissue, bodily fluids, or ingested food [1]. *Dermatobia hominis* is an insect native to Central and South America that is known to parasitize its hosts by laying eggs on blood-sucking arthropods such as mosquitos or ticks. The larvae from these eggs can then enter the skin of humans or mammals through an arthropod bite wound or by burrowing through a hair follicle to subcutaneous tissue [2]. The larvae remain in the subdermal tissue between 27 and 128 days, after which the adult larvae drop to the ground and pupate for 27 to 78 days before maturing into adult botflies [3]. Botfly larvae possess a row of concentric, backward-facing spines as well as two posterior respiratory spiracles that lie flush with the host's skin to allow for adequate breathing [1,2].

The typical lesion associated with botfly myiasis is an erythematous, raised, furuncle-like lesion with central necrosis most commonly affecting the limbs [3]. Common symptoms associated with the skin lesions include itching, sensation of moving, and occasional lancinating pain [4]. Because of the low incidence in the United States and non-specific symptoms, the diagnosis of botfly myiasis is often missed and treatment is often delayed [3]. Complete blood count might sometimes show elevated levels of leukocytes and eosinophils due to local inflammatory responses to infestation, however the diagnosis is confirmed only on identification of larvae [3].

For cases of botfly myiasis that present atypically, imaging is a useful modality to visualize larvae prior to more invasive treatments [5]. A prior study has shown that use of Doppler ultrasound with a high-resolution (10-MHz) soft-tissue transducer allowed for visualization of the larvae to confirm myiasis more rapidly and with high sensitivity and specificity [6]. Use of such imaging might allow for more rapid diagnosis of botfly myiasis and help avoid confusion with other similarly presenting dermatologic conditions.

## Case presentation

**Figure 1:**
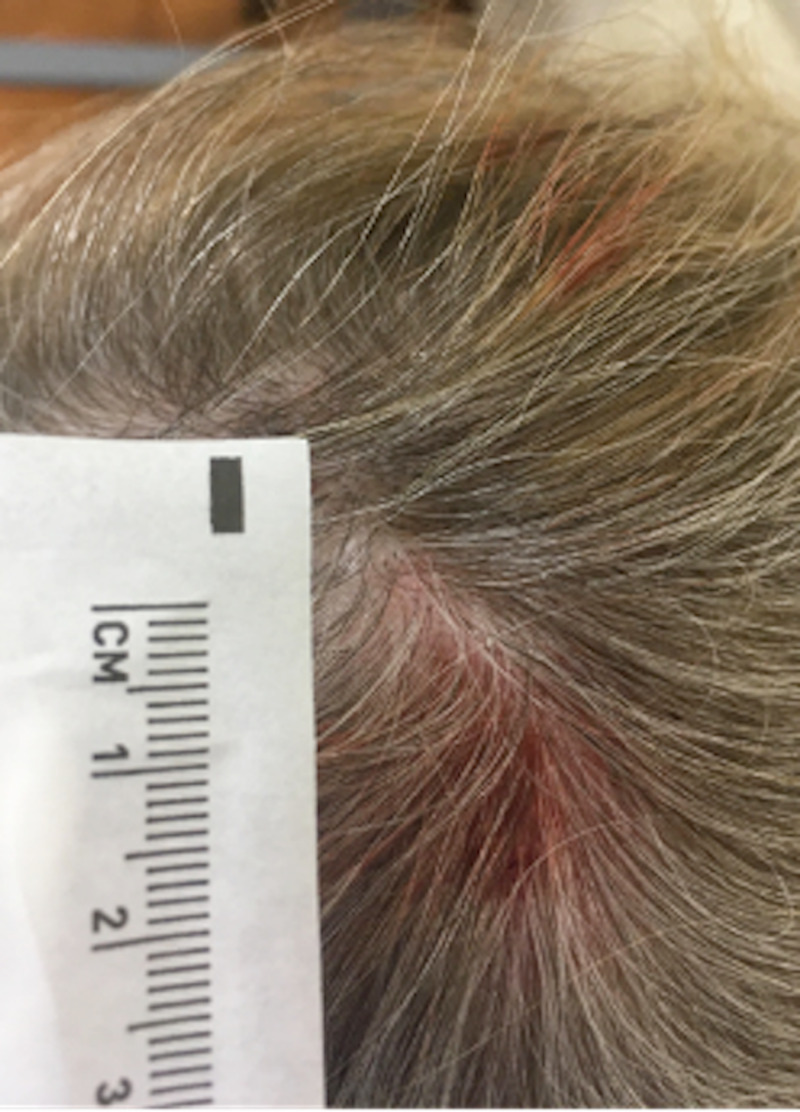
A 2 cm erythematous patch was located on the posterior scalp with no identifiable central necrosis.

**Figure 2:**
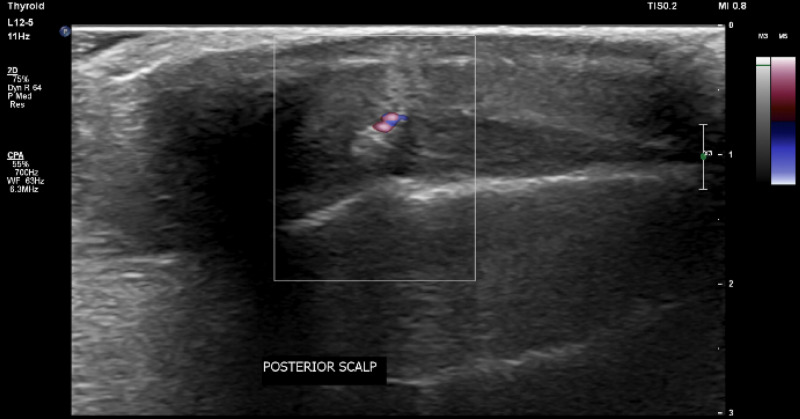
Hyperechoic region indicative of infestation with live botfly larva as evidenced by vascularity on doppler and movement on cine images. There is edema of the adjacent subcutaneous tissues with no sonographic evidence of abscess.

**Figure 3:**
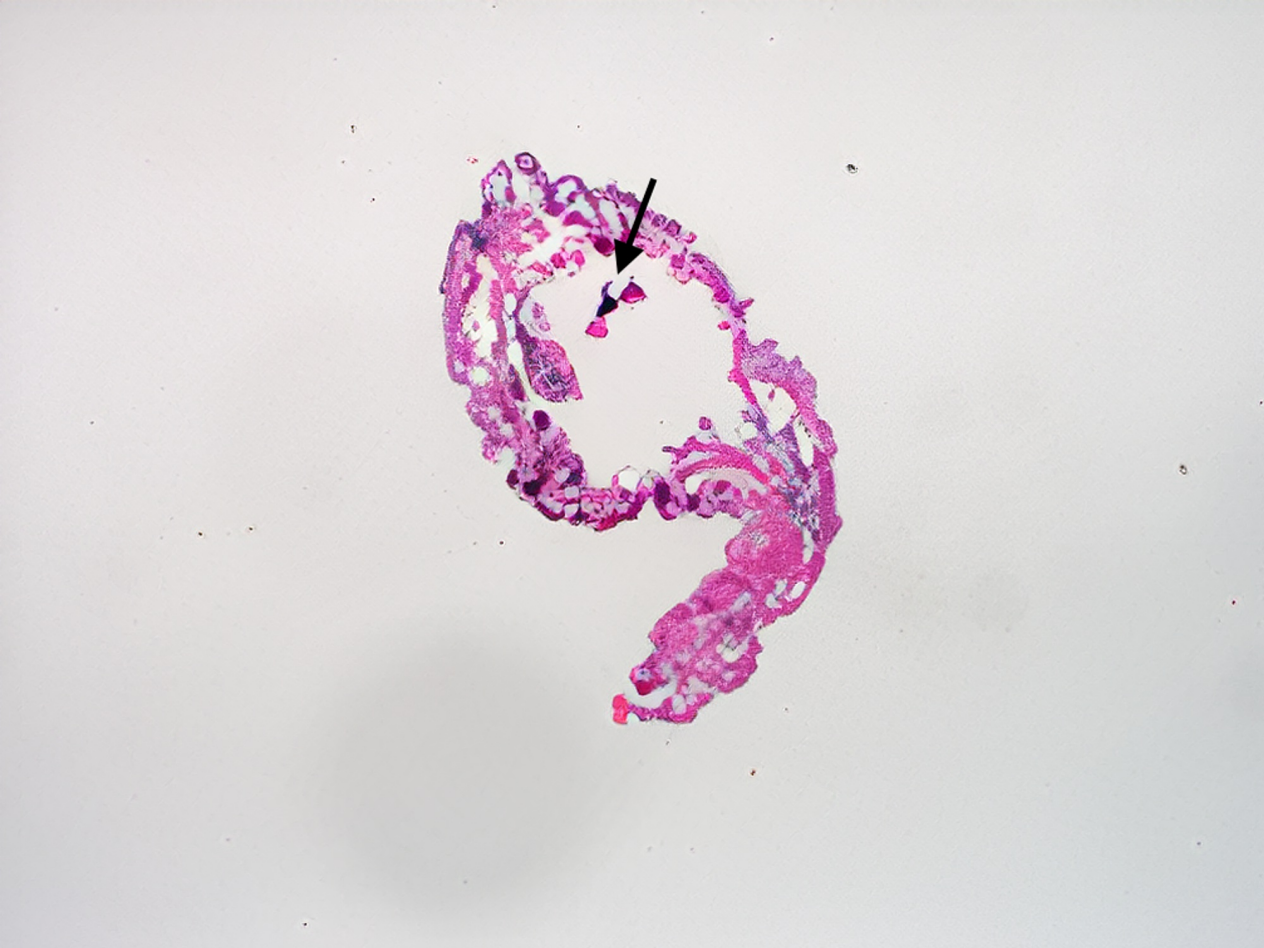
On pathologic evaluation following removal, the specimen consisted of a larva with relatively thick cuticle covered with spines. A pigmented posterior respiratory spiracle (arrow) was noted, consistent with a Dermatobia hominis larva.

**Figure 4:**
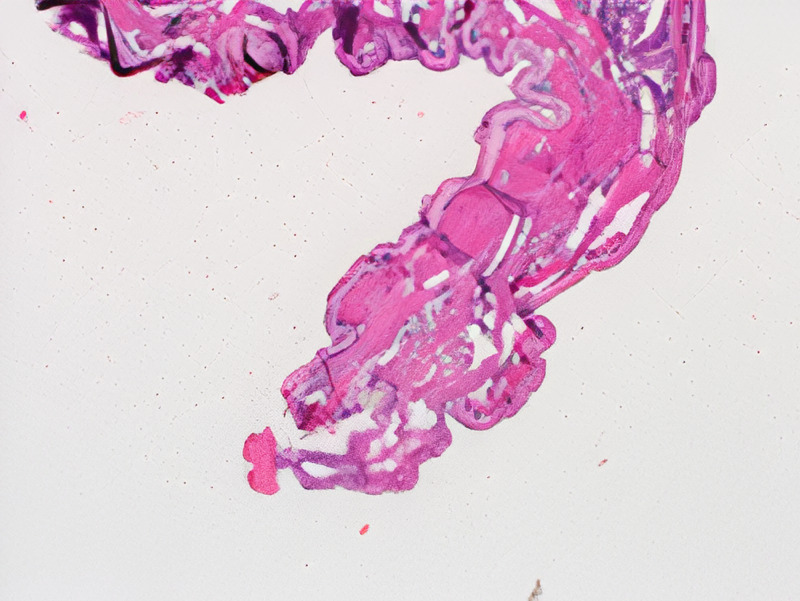
Striated muscle was seen directly under the cuticle.

The patient is a 50-year-old female who presented to a University of Florida Internal Medicine outpatient clinic in Gainesville, Florida. She had been conducting research in the rain forest on the border of Brazil and Bolivia and believed she was bitten by an insect on her posterior scalp. She returned to the United States and presented to the clinic 10 days after the reported insect bite. She experienced a skin lesion with itching in the area of her insect bite and reported that she could feel a moving sensation beneath the skin lesion. She denied any fever, chills, malaise, or headaches. A 2 cm erythematous patch was visualized on the posterior scalp (Figure 1), but efforts to find an opening and remove the larvae in clinic were unsuccessful. An ultrasound with doppler of the soft tissues in the scalp was ordered to attempt to visualize the larvae (Figure 2) and a general surgery appointment was made for removal. The surgical specimen was examined by pathology (Figure 3, Figure 4) and determined to be a botfly larva.

## Discussion

While human botfly myiasis can sometimes be identified by pathognomonic skin findings and associated symptoms, atypical presentations might require further investigation to confirm the diagnosis prior to surgical removal. Here we presented a patient whose associated skin lesion lacked the typical characteristics of induration and a central punctum, and Doppler ultrasound was a useful, non-invasive tool to confirm our suspicion for larval infestation. We believe that in cases where a patient has travelled to a botfly endemic area and presents with symptoms concerning of myiasis, ultrasound should be used. It has been used and proven to be an effective method of examination [7]. As ultrasound equipment becomes readily available, its use may help to confirm clinical suspicion in patients that refer to a moving sensation in their skin lesions. The ultrasound examination shows the shape and segmentations of the larvae in addition to the larva body spiracles and flow of larval body cavity fluid [7].

Our patient was treated with surgical removal of the botfly larvae with local anesthesia and wound debridement, which is the ideal treatment of botfly myiasis when available. However, in regions where medical resources are scarce, there are alternative treatments that can be used for larval removal. The larvae can be deprived of oxygen via application of occlusive substances such as petroleum jelly, nail polish, or plant extracts over the wound, causing the larvae to emerge from the wound for air to be removed with tweezers [8]. Alternatively, lidocaine can be injected into the base of the lesion to force the larvae out of the central punctum via a buildup of pressure [9]. Attempting to directly remove the larvae from the central punctum should be avoided as the larvae have a tapered shape with a row of spines that prevents simple extraction, and doing so might result in retained portions of the larvae leading to further infection and inflammation [3].

## Conclusions

Identification of botfly myiasis and proper treatment are crucial for timely and proper care of patients who become infected. When appropriate resources are available, they should be utilized to more competently and comprehensively provide patients with the care that they need. In the appropriate clinical setting, Doppler ultrasound is an excellent method to confirm the diagnosis of botfly myiasis, prevent misdiagnosis, and avoid delayed treatment.

## References

[1] Hohenstein EJ, Buechner SA (2004). Cutaneous myiasis due to Dermatobia hominis. Dermatology.

[2] Parsh S, Parsh B (2019). Treating parasitic human botfly. Nursing.

[3] Shenouda M, Enten G, Nguyen T, Mangar D, Camporesi E (2018). Human botfly: a case report and overview of differential diagnosis. J Investig Med High Impact Case Rep.

[4] O’Donel A (1984). Cutaneous myiasis. Arthropods and Human Skin.

[5] Bowry R, Cottingham RL (1997). Use of ultrasound to aid management of late presentation of Dermatobia hominis larva infestation. J Accid Emerg Med.

[6] Quintanilla-Cedillo MR, León-Ureña H, Contreras-Ruiz J, Arenas R (2005). The value of Doppler ultrasound in diagnosis in 25 cases of furunculoid myiasis. Int J Dermatol.

[7] Richter J, Schmitt M, Müller-Stöver I, Göbels K, Häussinger D (2008). Sonographic detection of subcutaneous fly larvae in human myiasis. J Clin Ultrasound.

[8] Platt SG, Schmidhauser CA, Meerman JC (1997). Local treatment of human botfly myiasis in Belize. Econ Bot.

[9] Lebwohl MG, Heymann WR, Berth-Jones J, Coulson I (2006). Myiasis. Treatment of Skin Diseases. Comprehensive Therapeutic Strategies.

